# Insights into the Preparation of and Evaluation of the Bactericidal Effects of Phage-Based Hydrogels

**DOI:** 10.3390/ijms25179472

**Published:** 2024-08-30

**Authors:** Mengyuan Gao, Yuhan Wang, Hanyue Zhuang, Yanxia Zhu, Na Chen, Tieshan Teng

**Affiliations:** School of Basic Medical Sciences, Henan University, Kaifeng 475004, China10190136@vip.henu.edu.cn (N.C.)

**Keywords:** bacteriophage (phage), hydrogel, antibacterial, wound healing

## Abstract

The rise of antibiotic-resistant strains demands new alternatives in antibacterial treatment. Bacteriophages, with their precise host specificity and ability to target and eliminate bacteria safely, present a valuable option. Meanwhile, hydrogels, known for their excellent biodegradability and biocompatibility, serve as ideal carriers for bacteriophages. The combination of bacteriophages and hydrogels ensures heightened phage activity, concentration, controlled release, and strong antibacterial properties, making it a promising avenue for antibacterial treatment. This article provides a comprehensive review of different crosslinking methods for phage hydrogels, focusing on their application in treating infections caused by various drug-resistant bacteria and highlighting their effective antibacterial properties and controlled release capabilities.

## 1. Introduction

The human body hosts numerous benign colonizing bacteria in areas such as the gut and skin, which play a crucial role in external communication. Nevertheless, when these bacteria migrate to sites like the lungs or bladder, they can readily lead to bacterial infections [1]. Antibiotics, traditional drugs used for treating or preventing bacterial infections [2], have significantly contributed to improving human health. However, their excessive use and misuse have led to bacterial resistance, resulting in the emergence of multidrug-resistant (MDR) strains [3]. Infections caused by MDR microorganisms can be exceptionally challenging to treat, leading to prolonged treatment times [4], mortality rates among patients, and a heightened economic burden of treatment [5], posing a significant threat to the global economy [6]. While new antibiotics hold the potential for controlling multidrug-resistant bacteria [7], their development has slowed due to cost and market profitability pressures, creating a pressing need for new antibacterial therapies [8]. In recent years, bacteriophage therapy has successfully treated life-threatening multidrug-resistant bacterial infections [9], providing a potential alternative to antibiotics for treating bacterial infections [10]. Phage therapy offers significant advantages, including more universal applications, host specificity, and broader antibacterial potential, and causes less harm to the human body compared to traditional antibiotics [11].

The formulation of phages presents a dual challenge: ensuring both the biostability of the phages and the physical stability of the formulation (e.g., solution, suspension). For phages to become viable therapeutic products, their native structure and biological activity must be preserved during production and storage [12,13]. Currently, there are ongoing issues with the clinical use of phage-based solutions or suspensions, such as the inability to localize free phages at the target site, insufficient effective residence time, narrow host-range and suboptimal antibacterial efficacy [14,15]. To date, the development of stable phage formulations for therapeutic purposes remains an underexplored area of research. Some previous reports on phage delivery systems, including nanocarriers and liposomes, have indicated limited applicability due to their sensitivity to external physical and chemical factors, such as temperature and osmotic pressure [16]. Hydrogels offer a remarkable array of functional advantages, including high water absorption, biocompatibility, biodegradability, and efficient material delivery. They also exhibit antibacterial properties, adhesion, and hemostatic capabilities, as well as anti-inflammatory and antioxidant effects. Furthermore, hydrogels possess self-healing properties, conductivity, and wound monitoring capabilities [17]. Owing to their diverse set of attributes, hydrogels are outstanding candidates for phage delivery and are extensively utilized in the biomedical field, including in applications such as wound dressings, oral gels, implantable devices, hydrogel microneedles, and injectable hydrogels [18]. This review centers on infections caused by diverse drug-resistant bacteria and delves into key aspects of phage hydrogel therapy, including preparation, research model construction, and therapeutic effects under various bacterial infections. The aim is to broaden the conceptual framework for advancing phage therapy and to provide prospects for its future development and research.

## 2. Various Types of Hydrogels with Different Crosslinking Methods

Hydrogels are a type of three-dimensional network structure gel that swiftly expands in water and retains a substantial volume of water without dissolving. Hydrogels are commonly cross-linked using two strategies: physical crosslinking and chemical crosslinking (Table 1) [19].

### 2.1. Utilizing Physical Mechanisms for Crosslinking

Physical crosslinking methods for developing hydrogels encompass ionic crosslinking, thermal gelation, freeze–thaw, self-assembly, and other techniques. Polymers capable of forming hydrogels through ionic crosslinking include Alginate, PVA-SA, PolyHIPE/Nanocellulose, Hydroxyapatite (HA), and beta tricalcium phosphate (β-TCP). Yongsheng Ma and colleagues created a phage K-containing hydrogel by combining 2% (*w*/*v*) sodium alginate with 10^8^ PFU/mL phage K and crosslinking it in a 100 mM calcium chloride solution, which effectively boosts the survival of free bacteriophages in simulated gastric conditions [20].

An alternative method for creating hydrogels through physical crosslinking involves utilizing thermal gelation. Poloxamer 407 (P407), also known as Pluronic F-127, is a tri-block copolymer composed of polyethylene oxide (70%) and polypropylene oxide (30%). At lower temperatures (typically <15–25 °C), P407 remains in a liquid state, enabling the loading of therapeutics for subsequent release when it transitions into a gel state. Previously, P407 has been explored as a carrier for *E. faecalis* phages, *A. baumannii* phages, and antibiotics such as vancomycin, levofloxacin, and metronidazole [21].

Additionally, the freeze–thaw method acts as a physical crosslinking technique for the formation of hydrogels, and Polyvinyl Alcohol (PVA) is frequently chosen as the polymer for this process. PVA is a hydrogel feedstock polymer known for its semi-crystalline structure and repetition of isomers (CH_2_CHOH). Its high content of hydroxyl groups promotes water absorption and expansion of the polymeric network, facilitating drug release. Scarlet Milo and her colleagues created a 10% *w*/*v* PVA solution containing phages by dissolving and cooling 20% *w*/*v* polyvinyl alcohol and then adding bacteriophage lysate at a 1:1 ratio. The resulting PVA solution was applied to the catheter, then frozen overnight at −20 °C to form the phage-containing PVA gel, effectively extending the blockage time and successfully eliminating bacterial colonization on the catheter [22].

Structured hydrogels made of self-organized M13 bacteriophage bundles were visible in electron micrographs in their cross-linked state, capable of absorbing up to 16 times their weight in water. These hydrogels showed advanced properties at room temperature, such as self-healing under biological conditions, auto fluorescence in three channels with decay through biodegradation, allowing non-destructive imaging, and bioactivity towards host bacteria in their cross-linked state. In 2021, Peivandi’s team successfully created a composite phage hydrogel using M13 phages and 0.25% (*w*/*v*) bovine serum albumin at a lower phage concentration, demonstrating remarkable properties including high water absorption, biological activity, adjustable mechanical properties, and self-repair capabilities [23].

### 2.2. Employing Chemical Agents for Crosslinking

Chemical crosslinking connects the network structure of hydrogels through chemical bonds, typically initiating polymerization primarily through crosslinking agents in an aqueous solution, which allows monomer molecules in the hydrogel to form a cross-linked structure.

PEG-4-MAL is commonly utilized in Michael-type addition. Wroe et al. [24] first combined an adhesive peptide and a crosslinking agent in a buffer, followed by the addition of 1.2 × 10^8^ PFU/mL of active phages. The resulting phage mixture, combined with 4.0% (*w*/*v*) PEG-4-MAL macromers (20 kDa) at a pH range of 6.0–6.5, forms a phage hydrogel capable of effectively controlling orthopedic-related infections. Susan M. Lehman and her team utilized PEG-polyurethane-coated catheters derived through bulk polymerization and subsequently pretreated the hydrogel catheters with phages. Following assessment with a multi-day continuous flow in vitro model in urine, it was observed that the phage-treated hydrogel catheters demonstrated a significant reduction in bacteria and biofilm formation [25]. Under the leadership of Mayhar Bassi [26], a team blended a 2% chitosan (CS) solution with a 5% starch solution and 2.5% glutaraldehyde, after which the mixture was transferred to a mold and incubated at −80 °C for 24 h. This hydrogel demonstrated outstanding biocompatibility, biodegradability, and effective slow controlled release properties.

Chemical crosslinking for preparing hydrogels offers several advantages, like achieving permanent crosslinking due to covalent bonds, which results in higher mechanical strength [27]. Fixed crosslinking agents also allow for a controllable crosslinking density [28]. However, it has drawbacks, including toxicity of the agents, which limits usage [29], and a more complex preparation process compared to physical crosslinking [28]. The hydrogel preparation method should be selected based on the specific polymers, intended uses, application scenarios, and other relevant factors.

**Table 1:** Various types and characteristics of hydrogel crosslinking.

Crosslinking Type	Polymer	Preparation Method	Advantages	Ref.
Physical crosslinking	Alnigate	Ionic crosslinking	Excellent biocompatibility, low viscosity	[20,30]
	PVA	Freezing and thawing	Biocompatible, film-forming ability, chemical stability	[31,32]
	PCL-Col I nanofibers	Thermal gelation	Adjustable mechanical strength, highly hemostatic	[33]
	HA/β-TCP	Ionic crosslinking	Similar in structure and composition to bone minerals	[34,35]
	Eudragit^®^ S100 and Alnigate	Physical crosslinking	Excellent compactness	[22,36]
	PolyHIPE/Nanocellulose	Ionic crosslinking	/	[37]
	PVA-SA	Ionic crosslinking	Strong hydrophilicity, painless removal	[31]
	Agarose/HAMA	Thermal gelation	Thermal reversibility, low cell adhesion	[38]
	PNIPAMco-ALA	Thermal gelation	Thermal reversibility, hardly degradable	[39]
	HPMC	Thermal gelation	Thermal reversibility, biodegradability	[40,41,42]
	QCS poly (xylitol sebacate)–APP	Freeze-thawing	Biodegradability Low toxicity, biocompatibility	[43]
	Agarose	Physical crosslinking	Withstands acidic conditions, biodegradability	[44]
	Chitosan	Ionic crosslinking	Biocompatibility, biodegradability, non-toxicity	[45]
	Poloxamer P407	Thermal gelation	Good bactericidal effect, thermo-reversible properties	[46]
	Pluronic^®^ F-127/HPMC	Thermal gelation	Thermal responsiveness,	[47]
Chemical crosslinking	PEG-polyurethane	Bulk polymerization	heat reactivity Anti-biological pollution, hardly degradable	[25,48,49,50]
	PEG-4MAL	Michael-type addition	Biodegradability	[24]
	CS-NP	Coercavation method	/	[51]

## 3. Enhancing Phage Hydrogel Therapy for Treating Infections Caused by a Range of Bacterial Strains

### 3.1. Phage Hydrogel Therapy for Infections Caused by E. coli

*E. coli* is a conditional pathogenic bacterium that can cause local tissue or organ infections in humans or animals under certain conditions. With the use of antibiotics, large numbers of drug-resistant bacteria are appearing, and phage hydrogel is an effective antibacterial material that can be used as a reliable therapeutic method. The following describes the corresponding phage hydrogel therapies for different serotypes of *E. coli* (Table 2). Han-Yu Shen et al. [52] targeted local infection of *E. coli* DH5α by embedding the targeting phage HZJ into an alginate hydrogel sample through physical crosslinking, creating a phage-based hydrogel wound dressing. The hydrogel released 10% of the phage within 24 h, and the number of bacteria killed reached 57% to 67% (*p* < 0.001) within 2 h, with antibacterial effects lasting at least 24 h. Tricalcium phosphate (TCP) is frequently utilized as a prosthetic material for bone substitution in the treatment of osteoarticular diseases and injuries. R. Ismail and his colleagues found that the incorporation of alginate hydrogels loaded with phage λvir as a coating on TCP ceramic bone substitutes could effectively delay the process of phage desorption, resulting in a prolonged release of the phage. The results demonstrate that incorporating an alginate hydrogel over TCP ceramic pellets increases the initial phage concentration on the material and extends the release time of phages to two weeks compared to control pellets. Moreover, these alginate-coated biomaterials exhibit accelerated bacterial lysis kinetics, making them a promising choice for practical prosthetic devices in bone and joint surgeries by facilitating localized phage therapy for bacterial infections over an extended duration [34].

Antibacterial materials that are both effective and affordable have garnered significant interest within clinical wound care practices. Cheng’s team utilized electrospinning to combine phage T4 with polycaprolactone/collagen I (PCL-ColI) nanofibers, with the purpose of eliminating *Escherichia coli* infection while concurrently facilitating hemostasis. The PCL-ColI membrane incorporating T4 phage demonstrated exceptional antibacterial efficacy, with a rate of above 90%. In vivo testing revealed that the PCL-ColI B membrane fully degraded within eight weeks, and no apparent pathological reactions were observed in the muscle or subcutaneous layer tissues at the back of rabbit test subjects [33].

### 3.2. Bacteriophage Hydrogel Therapy for Infections Caused by S. aureus

Phage-based hydrogels have recently emerged as a promising therapy option for various infections caused by *S. aureus*, highlighting their exceptional efficacy in addressing this pathogen (Table 3). Prabhjot Kaur et al. [31] utilized a PVA-SA mixed hydrogel membrane that incorporated phage MR10 as a wound dressing for effectively targeting and treating burn wound infections caused by *S. aureus*. Using a mouse burn wound model, this study demonstrated that the phage-enhanced PVA-SA mixed hydrogel membrane not only developed a protective barrier for the burn wound but also created an essential moist environment for optimal tissue regeneration. Additionally, the membrane exhibited remarkable antibacterial efficacy, as evidenced by a significant reduction of approximately 6 log_10_ in *S. aureus* biomass. These findings underscore the membrane’s effectiveness in lowering bacterial levels and highlight its potential as a potent antibacterial protective shield.

Orthopedic implant infections are a prevalent concern in medical practice. Sandeep Kaur and his colleagues formulated MR-5 phages and Linazolamide-coated HPMC wires (dual-coated wires) to target infections caused by *S. aureus* ATCC 43300 [41]. In their research, an animal model was established wherein infection was induced in the mouse femoral joint, followed by the implantation of K-wires coated with both phage and linezolid (dual-coated wires) into the femoral medullary canal. Mice implanted with dual-coated wires showed the most substantial decrease in bacterial adhesion and joint inflammation, and faster recovery of limb movement and motor function. Furthermore, none of the treatments led to the emergence of resistant mutants.

Beyond its impact on healthcare, a *S. aureus* infection also represents a substantial food safety threat. Using freeze–thaw technology, Reuben Wang’s team designed a positively charged Quaternized Chitosan (QCS) hydrogel that integrates bacteriophage 44AHJD and is cross-linked with a multivalent agent, aiming to improve its effectiveness [43]. The hydrogel phage controlled release model can release up to 60% of phage particles within a 6 h timeframe, effectively combating the proliferation of bacteria and preventing the development of phage-resistant strains.

### 3.3. Bacteriophage Hydrogel Therapy for Infections Caused by P. aeruginosa

*P. aeruginosa* has the potential to induce infections in multiple areas of the body, encompassing the respiratory tract, urinary tract and soft tissues, and is linked with severe conditions such as pneumonia, bloodstream infections, and wound infections. In the following section, we present the antibacterial effects of phage hydrogels against infections caused by *P. aeruginosa* (Table 4). W. A. Sarhan’s team added bee venom (BV) to honey/polyvinyl alcohol/chitosan (HPCS) nanofibers to produce HPCS-BV, and then loaded phage PS1 into HPCS-BV for the treatment of multidrug-resistant *P. aeruginosa* wound infection. The HPCS-BV/PS1 nanofibers demonstrated significant antibacterial efficacy against drug-resistant *P. aeruginosa*, reducing the initial count from 7 × 10^8^ CFU/mL to nearly 0 within 24 h, highlighting their effectiveness against resistant strains [57].

G. R. Abdellatif and colleagues conducted a study exploring the implementation of phage vB_Pae_SMP1/SMP5 combined with carboxymethylcellulose hydrogel for managing burn wound infections induced by carbapenem-resistant *P. aeruginosa* (CRPA) [58]. Inhibition zones were detected around the cups containing either of the tested hydrogels of SMP1 or SMP5, whereas no inhibition zone was observed around the cups with the control hydrogel. To evaluate the therapeutic efficacy of phage-incorporated hydrogel against CRPA, they established a mouse burn wound model infected with CRPA. The findings revealed a 60% survival rate in the control group that received the hydrogel treatment, while the phage-containing hydrogel treatment group achieved a 100% survival rate, demonstrating a superior anti-CRPA infection effect.

James A. Wroe and his team created an injectable hydrogel that can encapsulate phages and transport them to the area of bone infections. The release rates of phages from the hydrogel can be controlled by adjusting the gel formulation. These engineered hydrogels containing phages successfully eradicated their target bacteria in both planktonic and biofilm states without affecting the metabolic function of human mesenchymal stromal cells. These engineered hydrogel demonstrated a significant 4.7-fold reduction in live *P. aeruginosa* counts in murine radial segmental defects infected with *P. aeruginosa*, affirming their potential for the treatment of local bone infections [24].

S. M. Lehman and colleagues introduced a method wherein PEG-polyurethane hydrogel-coated catheters, paired with a mixed bacteriophage cocktail, were combined to combat urinary catheter-induced *P. aeruginosa* infection. This approach led to a remarkable reduction in *P. aeruginosa* biofilm by 4 log_10_ CFU/cm^2^ within 48 h (*p* < 0.01), demonstrating excellent antibacterial efficacy [49].

### 3.4. Bacteriophage Hydrogel Therapy for Infections Caused by K. pneumoniae

*K. pneumoniae*, a prevalent and highly antibiotic-resistant Gram-negative bacillus, frequently emerges as a leading pathogen in hospital-acquired infections, particularly causing pneumonia and respiratory illnesses. Remarkably, multiple encouraging results from clinical trials underscore the potential efficacy of using phage-containing hydrogel as an effective treatment for *K. pneumoniae* infections, providing optimism for addressing the challenges posed by antibiotic resistance (Table 5). Seema Kumari et al. [42] conducted a study in this field, wherein they developed a phage kpn5-HPMC hydrogel by encapsulating bacteriophage Kpn5 within a hot-melted hydrogel composed of the natural polymer HPMC. Seema Kumari and her team demonstrated the significant therapeutic potential of phage kpn5-hydrogel in treating *K. pneumoniae* infection in mice with a notable 63.33% survival rate.in the group treated with phage kpn5-containing hydrogel, compared to 0% in untreated counterparts by day 7 post-infection. Subsequently, the same team incorporated bacteriophages into a hydrogel composed of PVA-SA, aiming to evaluate the excipient’s therapeutic potential in treating wound infections caused by *K. pneumoniae*. Prabhjot Kaur et al. proposed an innovative approach by combining phages with hydrogels to effectively combat *K. pneumoniae* wound infection [31]. In their study, they utilized a PVA-SA mixed polymer ions cross-linked with targeted phage Kpn5 to create a phage-based PVA-SA hydrogel. The bacterial growth inhibition tests in vitro revealed a substantial 6.37 log_10_ reduction in *K. pneumoniae* biomass when exposed to Kpn5 phage PVA-SA hydrogel treatment, emphasizing the encouraging potential of this groundbreaking therapeutic strategy.

### 3.5. Bacteriophage Hydrogel Therapy for Infections Caused by P. mirabilis

*P. mirabilis* is a common pathogen in urinary tract infections, especially in individuals with urinary catheters or structural urinary tract abnormalities, as its swarming motility facilitates ascension and colonization of the bladder and kidneys. Moreover, the emergence of drug-resistant bacteria has presented substantial obstacles in managing *P. mirabilis* infections. In response to this challenge, numerous scholars have conducted research on phage hydrogels to develop innovative solutions for *P. mirabilis* infections (Table 5). A team led by Scarlet Milo devised a dual-coated catheter system [22], in which a PVA hydrogel envelops the phage in the lower layer and EUDRAGIT^®^S 100 (ROHM Co., Ltd., Evonik, Germany) in the upper layer on the catheter surface. Due to production of the bacterial urease enzyme by *P. mirabilis*, the urinary pH is elevated above 7, causing the upper gel to dissolve and subsequently releasing bacteriophages from the lower gel into the environment. The in vitro bladder model system demonstrated that the double-coated catheter effectively decreased the concentration of *P. mirabilis* by 6 log within 2 h and extended the catheter obstruction time from 13 h to 26 h, effectively delaying catheter blockage. Similarly, Lehman, S. M., et al. [25] engineered a catheter with a hydrogel embedding multiple bacteriophages tailored to target *P. mirabilis*. Subsequently to immersion in an artificial urine medium (AUM), the results exhibited a notable reduction of over 2 log_10_ CFU/cm^2^ in the *P. mirabilis* biofilm within 48 h.

### 3.6. Bacteriophage Hydrogel Therapy for Infections Caused by A. baumannii

*A. baumannii*, a Gram-negative coccobacillus commonly found in the environment, exhibits resistance to key antibiotics like colistin, tigecycline, and carbapenems, making it a significant nosocomial ESKAPE pathogen. The WHO has identified it as a critical research priority, while the CDC considers it an urgent public health threat. Fortunately, recent studies have demonstrated that phage hydrogels possess a potent antibacterial effect against resistant *A. baumannii* bacteria (Table 5). In previous research, Wei Yan and colleagues assessed the effectiveness of a phage-loaded thermosensitive hydrogel in treating wound infections caused by MDR *A. baumannii* [60], with IME-AB2 phage and MDR-AB2 serving as the model phage and bacteria, respectively. The IME-AB2 phage exhibited excellent storage stability in a ~18 wt% Poloxamer 407 hydrogel solution, with negligible titer loss over 24 months at 4 °C, and demonstrated sustained release with a cumulative release of 60% within the first 24 h. Their findings revealed that the IME-AB2 phage-embedded hydrogel significantly decreased *A. baumannii* levels by more than 5 log_10_ CFU/mL at 37 °C and effectively eliminated biofilms by 59%. Furthermore, in an in vitro wound infection model on pig skin, the IME-AB2 phage-embedded hydrogel demonstrated a remarkable ability to reduce bacterial counts by 90%. In 2023, the phage IME-AB2 combined with colistin was incorporated into a composite hydrogel composed of Pluronic^®^ F-127 (Sigma-Aldrich, St. Louis, MO, USA) and HPMC [47].The engineered hydrogel infused with phage IME-AB2 demonstrated excellent antibacterial efficacy by effectively eliminating bacteria in both planktonic (by 5.66 log) and biofilm (by 3 log) states while also inhibiting bacterial regrowth. Furthermore, this phage-loaded hydrogel proved highly effective in reducing 4.65 log of *A. baumannii* in a pig skin wound model.

**Table 5:** Phage hydrogel therapy for *K. pneumonia*, *P. mirabilis* and *A. baumannii* infection.

Strain	Phages	Polymer	Advantages	Preparation Method	Type of Infection	Antibacterial Effect	Ref.
*K. pneumoniae* B5055	Kpn5	HPMC	Thermal reversibility, biodegradability	Thermal gelation	Burn wound infection	The survival rate was 63.33% for mice	[42]
Carbapenem- resistant *K. pneumoniae*	Kpn5	PVA-SA	Strong hydrophilicity	Ion crosslinking	Wound infection	Decreasing the biomass of *K. pneumoniae* by 6.37 log_10_	[31]
*K. pneumoniae* B5055	Kpn5	HPMC	Thermal reversibility, biodegradability	Thermal gelation	Burn wound infection	The survival rate was 66.66% for mice	[40,61]
*P. mirabilis*	B4	PVA-Eudragit^®^ S 100	Poor biodegradability, poor cell adhesion	Freezing and thawing	Urinary catheter infection	Reducing *P. mirabilis* biofilm by 6 log	[22]
*P. mirabilis*	ΦPmir1/32/34/37	PEG-polyurethane	Heat reactivity, anti-biological pollution, poor biodegradability	Bulk polymerization	Urinary catheter infection	Reducing the number of *P. mirabilis* in biofilm by >2 log_10_ CFU/cm^2^	[25]
*P. mirabilis* 13 HER1094	T4	PEGpolyurethane	Heat reactivity	Chemical crosslinking	Urinary catheter infection	Reducing *P. mirabilis* biofilm formation by 90%	[48]
*A. baumannii* MDR-AB2	IME-AB2 phage	P407	Thermo-reversible properties	Physical crosslinking	Wound infection	Reducing *A. baumannii* >5 log_10_ CFU/mL, eliminating biofilm by 59%	[60]
*A. baumannii*	AB140/50	Chitosan	Biocompatibility, biodegradability, non-toxicity	Ionic crosslinking	Wound infection	Completely eliminating *A. baumannii*	[45]
*A. baumannii*	Phage 53	Poloxamer P407	Thermo-reversible properties	Thermal gelation	Wound infection	Resulting *A. baumannii* by 3.62 log _10_	[46]
*A. baumannii*	vB_AbaM-IME-AB2	Pluronic^®^ F-127/ HPMC	Thermal responsiveness	Thermal gelation	Wound infection	Killing 5.66 log of *A. baumannii*	[47]

## 4. Novel Environmentally Responsive Hydrogel

In addition to the diverse characteristics of hydrogels described above, recent studies have highlighted a new range of environmentally responsive hydrogels. These include pH-sensitive, photosensitive, enzyme-sensitive, magnetically sensitive, and REDOX-sensitive hydrogels. Xiaoliang Qi et al. prepared pH sensitive hydrogels using a grafting copolymerization reaction, which could adjust the release rate of loaded insulin according to the pH value in the environment. The release rate of the hydrogel was 26.1% within 24 h at pH 1.2, and exceeded 50% within 6 h at pH 7.4 [62]. The team led by Xun Tong used halogen bonds and visible light-sensitive parts to jointly prepare a photosensitive hydrogel. The hydrogel could achieve a transition from solution to gel under blue light irradiation, while the opposite process occurs under green light irradiation [63]. Shalini V. Gohil et al. prepared injectable and biodegradable enzyme-sensitive hydrogel by enzymatic crosslinking. This hydrogel could be degraded by lysozyme to release the loaded protein, and the release rate could be adjusted by changing the degree of acetylation [64]. Magnetic sensitive hydrogels carrying paramagnetic iron oxide nanoparticles (SPIONS) can be remotely heated under the effect of an external magnetic field, and are expected to improve tumor treatment effects by combining hyperthermia, radiotherapy and chemotherapy [65]. The magnetic sensitive gel loaded with SPIONS designed by Samantha A. Meenach et al. achieved good application results and can selectively kill M059K glioblastoma cells [66]. Oxidation-reduction sensitive hydrogels can undergo a liquid gel transition based on changes in the external redox environment [67]. Itsuro Tomatsu’s team constructed an oxidation-reduction sensitive hydrogel using ferrocenecarboxylic acid (FCA), which appears gel-like in its reduced state and solution-like in its oxidized state [68]. Recent studies have highlighted the various potential applications of hydrogels, extending beyond traditional uses as wound dressings and oral particles to innovative new methods of application. Jeong Yeon Choi et al. developed a spray-type alginate hydrogel for burned wounds, which can facilitate rapid wound treatment, effectively promote wound healing and greatly reduce pain [69]. On the basis of microneedles as a new and powerful drug transmission system, Ming Ji developed a hydrogel microneedle, which can maintain drug activity while having a good curative effect in wound healing [70]. These developments open new avenues for more immense and profound applications, showcasing the potential for significant advancements in biomedical field.

## 5. Conclusions

Phage therapy exhibits robust antibacterial potential while minimizing adverse effects on the human body. A noteworthy advancement in this area is the use of hydrogels as a dependable carrier for phages, which efficiently regulates their release during local applications. This strategic method optimizes the phages’ activity and concentration, thereby enhancing their antibacterial effectiveness. As a result, this approach has significantly elevated the efficacy of treatments across a wide range of bacterial infections, yielding impressive outcomes.

The integration of hydrogels in phage therapy represents a synergistic innovation with considerable potential for future medical applications. By leveraging the unique properties of hydrogels, phages can be maintained in an optimal state, ensuring their viability and functionality over extended periods, which is crucial for effective treatment regimens. Furthermore, the hydrogel matrix can be engineered to respond to specific environmental triggers, allowing for controlled and targeted phage release in response to infection cues, which can enhance the therapeutic impact while minimizing off-target effects.

The versatility and biocompatibility of hydrogels also paves the way for innovative delivery methods and formulations, potentially revolutionizing the management and treatment of bacterial infections. For instance, hydrogels can be molded into various shapes and used in wound dressings, in implants, or as injectables. In this review, we explored a variety of hydrogel polymers, highlighted the effectiveness of phage hydrogels in treating diverse bacterial infections, and offered innovative ideas to enhance phage therapy. These advancements could significantly contribute to the field of molecular microbiology and potentially replace antibiotics in the treatment of various bacterial infections.

## Figures and Tables

**Table 2:** Phage hydrogel therapy for *E. coli* infection.

*E. coli* Strain	Phages	Polymer	Advantages	Preparation Method	Type of Infection	Effect	Ref.
*E. coli* DH5α	HZJ	Alnigate	Biocompatibility, biodegradability, ease of gelation	Ion crosslinking	Wound infection	Reducing bacterial numbers by 59.3–68.5%	[52]
*E. coli* O157:H7	UFV-AREG1	PVA	Biocompatibility, chemical stability	Freezing and thawing	Skin wound infection	Increasing bacterial inhibition zone	[53]
*E. coli* XL-1	T4	PCL-Col I nanofibers	Biocompatibility, biodegradability, good flexibility	Thermal gelation	Wound infection	Improving antibacterial effect by 90%	[33]
*E. coli* K12	λvir	Hydroxyapatite/β-TCP	Osteoinduction, biodegradability	Ion crosslinking	Infection during bone reconstruction surgery	Enhancing the release of phages	[34,35]
*E. coli*	ΦKAZ14	CS-NP	Biocompatibility, biodegradability, non-toxicity	Coercavation	Alimentary infection	Providing strong protection against ΦKAZ14 phages	[51]
*E. coli* EV36	K1F	Eudragit^®^ S100/ Alnigate	Dissoluble, excellent compactness	Physical crosslinking	Alimentary infection	/	[36]
*E. coli* K-12 MG1655	T7	PolyHIPE/Nanocellulose	/	Ion crosslinking	Alimentary infection	Shielding phages in acidic conditions and releasing them in alkaline conditions	[37]
*E. coli* ATCC 11303	T4 Coli-proteus	PEG-polyurethane	Heat reactivity, anti-biological pollution	Chemical crosslinking	Urinary catheter infection	Reducing the biofilm formation by 90%	[48]
*E. coli* O104:H4	H4	Alnigate	Biocompatibility, biodegradability, ease of gelation	Ion crosslinking	Food pollution	Reducing *E. coli* count by 1.3 log_10_ CFU/g	[54]

**Table 3:** Phage hydrogel therapy for *S. aureus* infection.

*S. aureus* Strain	Phage	Polymer	Advantages	Preparation Method	Type of Infection	Effect	Ref.
MRSA	MR10	PVA-SA	Strong hydrophilicity	Chemical/ionic crosslinking	Burn wound infection	Decreasing bacteria number from 8 to 2 log_10_ CFU/mL	[31]
*S. aureus* H560	ΦK	Agarose/ HAMA	Thermal reversibility, low cell adhesion	Thermal gelation	Wound infection	Enhancing bacteria-killing capability	[38]
*S. aureus* ST228	ΦK	PNIPAMco-ALA	Thermal reversibility, not readily degradable	Thermal gelation	Skin and soft tissue infection	Effectively lysing *S. aureus* at 37 °C	[39]
MRSA	MR5 MR10	Liposomes	Biodegradable, does not elicit an immune response.	/	Wound infection	Reducing bacterial load by 4 log CFU/mL	[55]
*S. aureus* ATCC 43300	MR-5	HPMC	Thermal reversibility, biodegradability	Thermal gelation	Orthopedic implant infection	No bacterial burden found on the wire	[41]
*S. aureus*	Phage K	Alginate	Withstands acidic conditions, biodegradability	Ion crosslinking	Alimentary infection	Improving the survival of free phages	[20]
*S. aureus* BCRC 13077	44AHJD	QCS/poly (xylitol sebacate)–co-APP	Biodegradability, low toxicity, biocompatibility	Freeze–thawing	Food contamination	Releasing up to 60% of phage particles within 6 h	[43]
*S. aureus*	Modified phage	Alginate	Biodegradability	Ion crosslinking	Bone-related infection	Reducing soft tissue infection	[30]
*S. aureus*	Phage K	Agarose/ HAMA	Thermal reversibility, nondegradable, low cell adhesion	Thermal gelation	Bone-related infection	/	[56]

**Table 4:** Phage hydrogel therapy for *P. aeruginosa* infection.

*P. aeruginosa* Strain	Phage	Polymer	Advantages	Preparation Method	Type of Infection	Effect	Ref.
*P. aeruginosa*	PS1	HPCS	/	/	Wound infection	Reducing the bacterial count from 7 × 10^8^ CFU/mL to 0	[57]
CRPA	vB_Pae_SMP1/SMP5	CMC	Odorless, non-toxic	Chemical crosslinking	Burn wound infection	100% survival rate for mice	[58]
*P. aeruginosa*	KT28, KTN and LUZ19	Agarose	Temperature response	Physical crosslinking	Wound infection	Effectively hindering biofilm formation	[44]
*P. aeruginosa*	ΦPaer4/14/22 and ΦW2005A	PEG-4MAL	Biodegradability	Michael-type addition	Orthopedic implant infection	Reducing the CFU amount of bacteria by 16.9 times	[24]
*P. aeruginosa* isolate (Paer09)	FJK, R9–30, KR3–15	Alginate	Biocompatibiliy, biodegradability, hypotoxicity	Thermal gelation	Infection by fracture	Decreasing bacteria in the soft tissue by 6.5-fold	[59]
*P. aeruginosa*	PA5	PVA-SA	Strong hydrophilicity	Chemical/ionic crosslinking	Burn wound infection	Reducing *P. aeruginosa* biomass by 4.6 log_10_	[31]
*P. aeruginosa*	Phage cocktail	PEG-polyurethane	Heat reactivity, anti-biological pollution	Bulk polymerization	Urinary catheter infection	Reducing the number of *P. aeruginosa* biofilm by 4 log_10_ CFU/cm^2^	[25]
*P. aeruginosa*	M4	PEG-polyurethane	Heat reactivity, Anti-biological pollution	Bulk polymerization	Urinary catheter infection	Reducing biofilm cells from 7.13 to 4.13 log_10_ CFU/cm^2^	[49]
